# Dialectical behaviour therapy skills reconsidered: applying skills training to emotionally dysregulated individuals who do not engage in suicidal and self-harming behaviours

**DOI:** 10.1186/s40479-020-0119-y

**Published:** 2020-01-30

**Authors:** Mary Kells, Mary Joyce, Daniel Flynn, Ailbhe Spillane, Aoife Hayes

**Affiliations:** 1 Cork Mental Health Services, Cork Kerry Community Healthcare, Health Service Executive, Inniscarraig House, Western Road, Cork, Ireland; 2 National Suicide Research Foundation, Western Gateway Building, University College Cork, Cork, Ireland; 3 Mental Health Services, Cork Kerry Community Healthcare, Health Service Executive, St Finbarr’s Hospital, Cork, Ireland

**Keywords:** Dialectical behaviour therapy, Skills training, Community settings, Effectiveness, Adults

## Abstract

**Background:**

Dialectical behaviour therapy (DBT) is an evidence-based intervention for borderline personality disorder (BPD) but is an intensive treatment with significant health service costs. Access to DBT can sometimes be restricted due to limited resources. Positive results have been reported for the use of DBT skills training (DBT-ST), one of the four modes of standard DBT, in the treatment of individuals with BPD who self-harm. This study evaluates DBT-ST for a subgroup of individuals attending community mental health services who may have a diagnosis of BPD (or emerging BPD traits) but who are not actively self-harming.

**Methods:**

Participants in this study were 100 adults attending community mental health services with a diagnosis of BPD, emerging BPD traits or emotion dysregulation who were not actively self-harming. The majority of participants were female (71%), aged 25–34 years (32%), single (48%) and unemployed (34%). Participants partook in a 24-week DBT-ST intervention delivered by DBT therapists. Outcome measures included the Difficulties in Emotion Regulation Scale (DERS), the DBT Ways of Coping Checklist (DBT-WCCL) and the Five Facet Mindfulness Questionnaire (FFMQ). Measures were administered at pre-intervention, at the end of each skills module, and at post-intervention.

**Results:**

Significant reductions in emotion dysregulation (DERS) and dysfunctional coping (DBT-WCCL) scores were reported from pre- to post-intervention (*p* < .001). A significant increase in mindfulness scores (FFMQ) and DBT skill use (DBT-WCCL) was also observed (*p* < .001). However, the drop-out rate was high (49% at post-intervention).

**Discussion:**

The results of this uncontrolled study suggest that a standalone 24-week DBT-ST intervention may have a beneficial impact in terms of a reduction in emotion dysregulation and dysfunctional coping, and an increase in mindfulness and DBT skills use in patients with BPD/ emerging BPD traits who are not currently engaging in self-harm. Adequately powered randomised controlled trials are required to determine treatment efficacy in comparison to standard DBT for this population.

## Background

Borderline personality disorder (BPD) is a complex and challenging mental health diagnosis, characterised by a pervasive instability in affect, identity, interpersonal relationships and dysregulated behaviour [1, 2]. While the population prevalence rates of BPD vary from 0.7% [3] to 2.7% [4], prevalence rates are as high as 10 and 20% in outpatient and inpatient populations respectively [5–7]. Suicidal behaviour is common in individuals with BPD, with at least 75% engaging in self-harm and approximately 10% dying by suicide [8]. However, not all individuals with BPD or severe emotion dysregulation actively engage in self-harm or other suicidal behaviours [9].

Dialectical behaviour therapy (DBT) has been recommended internationally as an evidence-based treatment of choice for individuals with BPD [10, 11]. In its standard form, DBT is a 12-month intervention comprising four modes of treatment: individual therapy, group skills training, phone coaching and team consultation [12]. Given the multiple modes of treatment, the time required for each on a weekly basis, and the overall duration of the programme, DBT is sometimes considered a time and resource intensive treatment [13]. Access to standard DBT is therefore often restricted due to limited resources and a shortage of DBT trained clinicians, particularly in public health services [9, 14].

In addition to patients with BPD who are actively self-harming and for whom DBT is indicated, there is also a subgroup of individuals who may have a diagnosis of BPD (or meet criteria for a diagnosis) who do not actively engage in self-harm. In our experience, in secondary care community mental health services, there is a real-world clinical need for treatment of these individuals who may have emerging emotion dysregulation difficulties but do not routinely engage in self-harming behaviours. Given the absence of ongoing behavioural dysregulation for this group of individuals, we considered the potential benefit of offering a DBT skills training (DBT-ST) programme, rather than the standard DBT programme with all four modes of treatment, to individuals with lower risk emotion dysregulation. This could also be a more economically viable way of providing an effective intervention to this client group.

DBT-ST as a standalone treatment has previously been evaluated for the treatment of individuals with BPD. Studies by Soler et al. [15], Linehan et al. [16] and McMain and colleagues [9] have evaluated the skills training component of DBT for its efficacy in treating BPD. While positive results have been reported for individuals who completed DBT-ST in all three studies, the samples differed to the target sample in the current study whereby individuals with BPD who self-harm were included in previous studies. Furthermore, while the same format of standard DBT-ST was followed in all three studies, the duration of the weekly skills training sessions and the overall treatment duration differed (e.g. 13 weeks in Soler et al. [15], 20 weeks in McMain et al. [9], and 1 year in Linehan et al. [16]). The client group we hoped to target in our service were individuals with BPD who weren’t actively self-harming. We therefore hypothesised that a DBT informed intervention where the skills are delivered weekly, exactly as per the standard DBT programme but over a shorter 24-week timeframe, would be sufficient to obtain positive outcomes for individuals. Unlike the standard DBT programme, we hypothesised that one delivery of the skills modules, rather than the additional repeating of modules, would be sufficient for this client group.

No study to our knowledge has been conducted on the effectiveness of a DBT-ST intervention for the above identified client group and over a 24-week treatment period. This study therefore aims to investigate the effectiveness of a 24-week DBT-ST intervention for individuals attending community services with either a diagnosis of BPD, emerging BPD traits or emotion dysregulation who are not actively self-harming or suicidal. Our a priori hypothesis was that DBT-ST would result in improved outcomes for participants in terms of reduced emotion dysregulation and dysfunctional coping, and increased mindfulness and DBT skills.

## Method

### Aim, design and study setting

The aim of this study was to examine the effectiveness of a 24-week DBT-ST programme for individuals with BPD or emerging emotion regulation difficulties, who were not routinely engaging in suicidal or self-harm behaviours. This was a quantitative study carried out in a secondary care community-based mental health setting in the Irish public health service. Self-report measures were administered to participants over the duration of the study. The DBT-ST programme was delivered at two sites where individuals from urban and suburban areas of Cork city attended community based mental health services. Recruitment of participants took place between March 2014 and August 2017. Ethical approval to conduct this research study was received from the Clinical Research Ethics Committee of the Cork Teaching Hospitals.

### Participants

The sample for this study were treatment seeking adults attending community mental health services in an urban area in the South of Ireland. Inclusion criteria were: a diagnosis of BPD or borderline personality traits^1^/significant emotion dysregulation^2^; a pervasive history of difficulties understanding and managing emotions; and demonstration of a commitment to participating in a 24-week DBT-ST programme. Exclusion criteria were: more than one incident of self-harm or suicidal behaviour in the past six months; an active psychosis (participants with a previous diagnosis of psychosis were not excluded); severe developmental delays, cognitive impairment or an intellectual disability (exceeding the mild range); and other diagnoses which might impact on engagement with group learning, and for which the evidence-based treatment should be prioritised (i.e. an Axis I diagnosis where the individual's difficulties were better explained by that diagnosis and for which there is an evidence-based treatment e.g. Social Anxiety requiring Cognitive Behaviour Therapy). The inclusion/exclusion criteria were reviewed by the referrer and the index community mental health team.

Participants had a diagnosis of BPD (DSM-IV-TR), had borderline personality traits or significant emotion dysregulation. Individuals with co-morbid axis I and II disorders and/or individuals on medication were also eligible for inclusion and were permitted in the study. Multi-disciplinary professionals on the community mental health team, under the lead of a Consultant Psychiatrist, completed the initial assessment with participants. This assessment was completed as part of a routine clinical evaluation and was not conducted specifically for the research study. If individuals met the above criteria, they were referred to the DBT team for potential inclusion in the DBT-ST programme.

Participants were referred to the DBT-ST programme by their community mental health team. Referral agents included consultant psychiatrists, community mental health nurses, occupational therapists, social workers and clinical psychologists. All referrals were initially reviewed by the DBT team to assess suitability with respect to eligibility criteria. Any queries regarding eligibility were discussed with the referral agent. Individuals then attended a clinical interview with a DBT therapist to determine if individuals met inclusion criteria. If criteria were met, the therapist oriented individuals to the programme and assessed future commitment to the programme. Suitable individuals were offered a place on the programme. Individuals who attended within the first three weeks of the intervention were recruited for study participation.

### Intervention

The intervention delivered was a 24-week DBT-ST programme. The DBT skills training component of standard DBT, as per Linehan [12], was delivered with the exception that the three modules were delivered once and not repeated, resulting in a 24-week programme. The programme comprised of mindfulness, distress tolerance, emotion regulation and interpersonal effectiveness skills which were organised into three modules [12, 17]. A breakdown of how the skills modules were structured is provided in table 1. Sessions were 2.5 h in duration and were delivered once a week either by one or two DBT therapists, depending on availability. The intervention was delivered by DBT therapists who had completed either Intensive or Foundational DBT Training with a licensed training provider. The therapists were members of DBT teams that were also delivering a standard DBT programme. Both teams were also availing of expert DBT supervision.

**Table 1:** Module content of DBT-ST programme

Module	Content
1	Two mindfulness and six distress tolerance skills training sessions
2	Two mindfulness and seven emotion regulation skills training sessions^a^
3	Two mindfulness and five interpersonal effectiveness skills training sessions

^a^At the outset of this study, all three modules were eight weeks in duration in line with standard DBT treatment recommendations [17]. However, following a published revision of the skills manual and consultation with the treatment developer (Prof. Marsha Linehan) in 2015, an additional emotion regulation session was added to module two and an interpersonal effectiveness session was removed from module three, resulting in 9 week and 7 week long modules respectively

### Measures

The following measures were completed at each time point:

- The Difficulties in Emotion Regulation Scale (DERS) is a 36-item self-report measure which assesses difficulties in emotion regulation across six subscales [18]. The internal consistency of the DERS in this study was 0.93.
- The Five Facet Mindfulness Questionnaire (FFMQ) is a 24-item self-report measure of mindfulness comprising five subscales [19]. The internal consistency of the FFMQ in the current study ranged from .73 to .91 on the different facets of the scale.
- The Dialectical Behaviour Therapy Ways of Coping Checklist (DBT-WCCL) is a 59-item self-report measure of skills used in response to difficult situations over the past month and has two subscales; DBT skills use and dysfunctional coping [20]. In the current study, the internal consistency of the DBT skills use subscale was 0.92 and 0.87 for the dysfunctional coping subscale.

The absence of self-harm behaviours was not formally measured over the course of the intervention. This was assessed by verbal self-report by participants. Individuals on the programme were encouraged to inform a member of their multi-disciplinary team or a DBT skills training facilitator if they had engaged in self-harm.

### Procedure

A group data collection session was scheduled to take place at the study sites at the start of the programme (and at the start of every module i.e. every 8 weeks when new participants joined the programme). If there were more than three participants starting at the beginning of a new module, a member of the research team attended the first group data collection session. For data collection points where there were less than three participants for the group data collection, the DBT therapist administered the measures on behalf of the research team. At pre-intervention, Participant Information Leaflets were distributed and participants were provided with an opportunity to ask questions about the research study prior to providing written consent for participation. Quantitative measures were then administered at five time points: pre-intervention (T1); end of module 1 (T2); end of module 2 (T3); post-intervention (T4); and 6 months post-intervention (T5). Measures at pre-intervention were completed at the beginning of the first skills group session. For subsequent time-points, data were collected at the end of the group session. Participants’ contact details were obtained at post-intervention to facilitate contact by the research team at follow-up. Participants were contacted by phone in advance of the follow-up data collection and were invited to complete the measures and return them to the research team by post within a two-week timeframe.

### Analysis

All self-report outcome measures were quantitative and were summarised by their mean and standard deviation. Independent sample ‘t’ tests were conducted to investigate potential differences between programme completers and drop-outs at baseline. For each outcome measure, multilevel linear mixed-effects regression models were used to estimate the mean at baseline (T1) and the mean change from baseline to each of the subsequent time-points. Mixed-effects models use all available data at each time-point rather than data from individuals assessed at all times. We included a random intercept in the models for the individual participants. These intercepts adjust for random heterogeneity in each outcome measure between subjects. Data were analysed using Stata version 13.1 and IBM SPSS Statistics 23.0 for Windows.

## Results

Complete diagnostic information was available for 100 participants. Fifty-nine participants had borderline personality traits only while the remaining 41 participants had a diagnosis of BPD. Just over half of the sample (55%) had a co-morbid diagnosis as noted on the referral form. The majority of participants were female (71%), aged between 25 and 34 years (32%), were single (48%) and unemployed (34%) (Table 2).

**Table 2:** Characteristics of sample

Characteristics	%
Sex (*n*=100)	
Male	29
Female	71
Age (*n*=100)	
18–24 years	22
25–34 years	32
35–44 years	20
45+ years	26
Marital Status (*n*=100)	
Single	48
In a relationship	28
Married	15
Separated/Divorced	9
Employment Status (*n*=100)	
Full-time	13
Part-time	18
Unemployed	34
Retired/Other/Homemaker	12
Student	12
Disability/Sick leave	11

### Drop-out and missing data

Data were available for 97 participants at T1, 87 at T2, 74 at T3, 51 participants at T4 and 26 participants at T5. Given the nature of this study, which was conducted in a publicly funded health service with limited resources, it was only possible to conduct data collection with those who completed the intervention. Drop-out in this study therefore refers to those who dropped out of the intervention. There was a 49% drop-out rate from treatment. There were no significant differences between treatment completers and drop-outs on baseline scores of self-report measures i.e. no difference in emotion regulation or dysfunctional coping scores.

There was also missing data at some time-points. In addition to missing data for participants who dropped out of the intervention, other reasons for missing data include participants not returning completed measures or completing measures outside of the two-week timeframe specified for data collection. Given the low return of data at T5 (26%), these data are not reported here. Change from pre-intervention to the subsequent three time-points will be detailed.

### Self-report measures

The results of the multilevel linear mixed-effects model analysis are presented in Table 3.

**Table 3:** Outcome Measure Estimated Means (M) and Confidence Intervals (CI) at Baseline and Each Subsequent Time Point

Variable	Estimate T1 M (95% CI) (*n* = 97)	Estimate T2 M (95% CI) (*n* = 87)	Estimate T3 M (95% CI) (*n* = 74)	Estimate T4 M (95% CI^a^) (*n* = 51)	% change T1 – T4
Dysfunctional Coping (DBT-WCCL)	45.74* (43.85, 47.63)	40.24* (38.11, 42.37)	36.14* (33.95, 38.33)	32.06* (29.65, 34.48)	- 30
DBT Skill Use (DBT-WCCL)	57.28* (53.48, 61.08)	74.14* (69.95, 78.34)	80.26* (75.87, 84.66)	85.55* (80.82, 90.28)	+ 49
Emotion Dysregulation (DERS)	128.83* (124.05, 133.60)	117.26* (112.04, 122.47)	99.76* (94.28, 105.24)	88.16* (82.05, 94.27)	−32
Nonacceptance	22.02* (20.81, 23.23)	20.12* (18.81, 21.43)	16.34* (14.96, 17.73)	14.41* (12.88, 15.94)	−35
Goals	19.98* (19.17, 20.79)	18.63* (17.73, 19.52)	16.74* (15.78, 17.69)	15.22* (14.17, 16.27)	−24
Impulse	21.01* (19.88, 22.15)	19.08* (17.85, 20.31)	16.07* (14.78, 17.36)	13.69* (12.30, 15.09)	−35
Awareness	19.90* (18.95, 20.85)	17.73* (16.69, 18.77)	15.29* (14.20, 16.39)	13.59* (12.40, 14.80)	−32
Strategies	29.20* (27.86, 30.54)	26.53* (25.07, 27.99)	19.66* (17.97, 21.36)	19.66* (17.97, 21.36)	−33
Clarity	16.58* (15.82, 17.34)	15.13* (14.30, 15.96)	13.34* (12.46, 14.21)	11.81* (10.84, 12.77)	−29
Mindfulness (FFMQ)	60.48* (58.09, 62.87)	67.86* (65.23, 70.48)	74.56* (71.79, 77.32)	80.76* (77.78, 83.74)	+ 34
Observe	12.42* (11.67, 13.17)	13.47* (12.67, 14.27)	14.74* (13.91, 15.56)	15.18* (14.30, 16.06)	+ 22
Describe	13.92* (13.21, 14.63)	15.18* (14.40, 15.95)	16.54* (15.73, 17.35)	17.88* (16.99, 18.76)	+ 28
Non-React	10.53* (9.87, 11.19)	12.97* (12.23, 13.71)	14.44* (13.66, 15.21)	15.82* (14.97, 16.67)	+ 50
Act Aware	12.07* (11.32, 12.82)	13.45* (12.62, 14.28)	14.44* (13.56, 15.32)	16.37* (15.41, 17.33)	+ 36
Non judge	11.61* (10.87, 12.35)	12.68* (11.85, 13.51)	14.05* (13.18, 14.92)	15.47* (14.50, 16.44)	+ 33

* = *p* < .001

^a^95% CI = 95% confidence interval

Linear mixed-effects models indicated significant changes from T1 to each subsequent time-point (T2, T3 and T4) on all outcome measures. Significant reductions were reported for dysfunctional coping and emotion regulation (*p* < .001). A significant increase in DBT skill use and mindfulness was also observed (*p* < .001). The percentage change from pre-to post-intervention (T1 to T4) ranged from 22 to 50%. The greatest change was observed for the Non-React subscale of the FFMQ (50%) with similar change observed in DBT Skill Use (49%). The smallest change was observed on the FFMQ ‘Observe’ subscale (22%).

## Discussion

The results of this study suggest that a standalone 24-week DBT-ST intervention may be beneficial for individuals with BPD/ emerging BPD who are not actively self-harming or engaging in other suicidal behaviours. A reduction in emotion dysregulation and dysfunctional coping, in addition to an increase in mindfulness and DBT skills use was observed from pre- to post-intervention. However, given the design of this study, the results ought to be interpreted with caution.

The provision of standard DBT in public community health services is both expensive and resource intensive. As a result, DBT is often a scarce resource which, by necessity, is ordinarily prioritised for individuals with the most severe presentations. This typically precludes individuals who primarily present with emotion dysregulation and are not currently suicidal or engaging in self-harm. Consequently, there is a significant clinical demand for adapted and less resource intensive programmes to meet the needs of such client groups. Findings from this study suggest that participation in a DBT-ST intervention may be appropriate for such individuals. Significant improvements were reported on all outcome measures from pre- to post-intervention. While data were collected for a subset of the sample at a 6-month follow-up time-point, participants numbers were small (*n* = 26) and thus were not considered robust enough to present here.

The dropout rate in the current study was quite high (49%) indicating that almost half of the sample did not complete the intervention. Analyses of data between individuals who dropped out of the programme versus programme completers found no significant difference between the groups on baseline scores of the self-report measures. A potential reason for the high dropout rate observed in this study is the lack of individual therapy or individual case management for participants during the intervention. Unlike other reported DBT-ST programmes where case management was provided (e.g. [16]), once participants began the DBT-ST intervention described in this study, they did not receive any further additional supports from the DBT team during the programme. In the study conducted by Soler et al. [15], lower drop-out rates from the DBT-ST intervention were observed (34%). However, the shorter duration of the intervention in the Soler et al. study may in part contribute to the higher retention rates observed. In order to fully understand the implications of this finding for clinical practice, we need to understand why individuals dropped out of this study. This could potentially be investigated in future studies by conducting qualitative interviews with participants who drop out of the intervention.

The findings from this study, which is the first of its kind to evaluate the effectiveness of a DBT-ST programme in routine clinical practice, corroborate some of the findings from randomised controlled trials (RCTs) conducted in this field. Given the difference in study design and sample composition, the results of the current study are not directly comparable with those of McMain et al. [9]. However, similar scores were observed on the DERS at baseline for participants in McMain et al.’s study [9] while participants in our study reported a greater reduction in emotion dysregulation at post-intervention. These findings offer promise for participants in terms of potential improvement in emotion dysregulation following participation in the intervention described here. The slightly longer duration of the intervention in our study (2.5 h × 24 weeks) in comparison to that described in McMain et al. [9] (2 h × 20 weeks) may also account for some of the variation in results between the two studies.

Analyses indicated that DBT-ST also had a positive impact on measures of mindfulness and emotion regulation which both improved over the course of the intervention. Outcomes on measures of mindfulness indicated a particular improvement for 'non-react' mindfulness, while 'non-acceptance' and 'impulse' subscales of the emotion dysregulation scale showed the largest decrease.

### Strengths and limitations

This study has several strengths. Given that participants in this study were not actively suicidal or engaging in self-harm, they would not have met the threshold to warrant a place on standard DBT in our local community mental health service. In this setting, resource limitations and the significant demand for the standard DBT programme results in those with the most severe levels of dysregulation and those who are suicidal and routinely engaging in self-harm behaviour being prioritised for standard DBT. The DBT-ST programme described here offered a briefer alternative intervention which focused on providing DBT-ST over a 24-week period in contrast to the longer 12-month standard DBT programme. The intervention was of a 24-week duration and delivered in line with the structure and content used in the first 6 months of skills training as per standard DBT [12]. This contrasts with adaptations to standard DBT-ST and varying treatment durations of skills training interventions reported to date [21].

While we obtained a relatively large sample size at the outset (*n* = 100 at baseline), which potentially results in a more precise mean, there was a high participant dropout over the course of the intervention. This may have inhibited the power of the analysis to detect a difference across the various time-points of the study. Nevertheless, as participants were referred to the programme by their community mental health team and subsequently attended a screening session with a member of the DBT team to assess suitability for the intervention, homogeneity with respect to eligibility criteria was ensured for this study.

Notwithstanding this, there are some limitations associated with the research reported here. Firstly, there was no control group, thereby limiting inferences that any positive changes reported directly arose as a result of the DBT-ST intervention. In addition, confounding effects may have arisen as it is possible that participants took part in other therapy offers whilst participating in the intervention described here. However, given the dearth of research conducted with this particular client group and in routine clinical practice, this study provides preliminary evidence to support the effectiveness of a standalone DBT-ST programme as a suitable intervention in a community setting for individuals with BPD/ emerging BPD.

Secondly, this study did not include a measure of borderline symptoms such as the Borderline Symptom Checklist (e.g. BSL-23). As there were a number of measures which addressed the primary outcomes for this research, we were conscious of not over-burdening participants with multiple assessment measures. Consequently, we cannot assume that participant’s borderline symptoms reduced following completion of the intervention. Although there is evidence to indicate that there was improved DBT skills use and mindfulness in addition to reduced levels of emotion dysregulation, further research is required to investigate if the intervention results in clinically meaningful change for participants in terms of symptomatology and co-morbid presentations such as depression and anxiety.

Thirdly, although data collection was attempted at a six-month follow-up, the return of completed measures was low (*n* = 26). Consequently, there were insufficient data to report here. The lack of sufficient follow-up data for analysis results in inconclusive findings regarding potential long-term gains for participants who complete the intervention.

Finally, it is acknowledged that a further limitation is the absence of adherence coding for DBT therapists providing the intervention. Financial constraints of conducting adherence coding in a publicly funded mental health service with limited resources resulted in this being beyond the scope of the current study. It is therefore possible that the results may not be generalisable; caution must be exercised when comparing the results of our studies to those reported in other studies. Arranging the funding for adherence coding is a priority for future research.

### Recommendations for future research

A RCT is required to determine treatment efficacy, in comparison to standard DBT, for the target population described in this study. If efficacy is shown, the standalone DBT-ST programme may be a cost and resource efficient alternative for health services as the intervention described here does not have the financial or labour burden associated with providing a standard DBT programme.

Similarly, in considering the economy of costs, patients could potentially undergo DBT-ST while awaiting access to standard DBT treatment. In this way, a change in symptoms may already have started during the wait-time for standard DBT, and for some patients, an intensive treatment may no longer be necessary. Alternatively, the subsequent treatment could potentially be shortened because patients will already have experience in applying the skills.

Another potential consideration in future research could be to implement DBT-ST proactively in order to prevent a more severe course in patients with significant emotion dysregulation and borderline traits.

Supplementary data analyses identified that DBT skill use was associated with decreases in DERS scores. These results suggest that skills use rather than factors such as group cohesion contributed to such improvements. Future research could measure the impact of group cohesion or other non-DBT skills related factors to confirm that the measured differences do in fact correlate with the intervention itself and not with other factors.

Given the promising findings from RCTs to date, further research studies are now warranted in routine clinical settings to investigate the potential effectiveness of DBT-ST for individuals who have a diagnosis of BPD and who self-injure in community settings. A comparison of DBT-ST, as described in this study, and standard DBT for individuals with BPD who self-harm would help to identify if DBT-ST is effective for this client group.

Given the relatively small number of participants at follow-up in this study, future research designs should ensure follow-up data collection is conducted with an adequate number of participants to provide robust information on the pattern of consistency/change following completion of the intervention.

## Conclusion

DBT-ST as a standalone treatment appears to be effective for individuals with BPD, emerging BPD traits or emotion dysregulation who are not actively self-harming. However, caution is warranted as the current study did not have a control or comparison group. Future controlled studies with other treatment options, as well as consideration of long-term gains, are required before definitive conclusions can be drawn about the effectiveness of DBT-ST for treating individuals with BPD/ emerging BPD traits.

## Data Availability

The datasets used and analysed in the current study are available from the corresponding author on reasonable request.

## Footnotes

1 In this study, individuals with BPD traits refers to individuals who the multi-disciplinary professional conducting the assessment perceived to have emerging BPD that warranted intervention but did not meet criteria for a BPD diagnosis at that time.

2 Emotion dysregulation in this study refers to significant emotion dysregulation that was not better explained by an Axis I diagnosis.
